# Nitric oxide signaling modulates synaptic inhibition in the superior paraolivary nucleus (SPN) via cGMP-dependent suppression of KCC2

**DOI:** 10.3389/fncir.2014.00065

**Published:** 2014-06-17

**Authors:** Lina Yassin, Susanne Radtke-Schuller, Hila Asraf, Benedikt Grothe, Michal Hershfinkel, Ian D. Forsythe, Cornelia Kopp-Scheinpflug

**Affiliations:** ^1^Division of Neurobiology, Department Biology II, Ludwig-Maximilians-University MunichPlanegg-Martinsried, Germany; ^2^Department of Physiology and Cell Biology, Faculty of Health Sciences, Ben-Gurion University of the NegevBeer-Sheva, Israel; ^3^Department of Cell Physiology and Pharmacology, University of LeicesterLeicester, UK

**Keywords:** nitric oxide, KCC2, post-inhibitory rebound, gap-detection, auditory brainstem

## Abstract

Glycinergic inhibition plays a central role in the auditory brainstem circuitries involved in sound localization and in the encoding of temporal action potential firing patterns. Modulation of this inhibition has the potential to fine-tune information processing in these networks. Here we show that nitric oxide (NO) signaling in the auditory brainstem (where activity-dependent generation of NO is documented) modulates the strength of inhibition by changing the chloride equilibrium potential. Recent evidence demonstrates that large inhibitory postsynaptic currents (IPSCs) in neurons of the superior paraolivary nucleus (SPN) are enhanced by a very low intracellular chloride concentration, generated by the neuronal potassium chloride co-transporter (KCC2) expressed in the postsynaptic neurons. Our data show that modulation by NO caused a 15 mV depolarizing shift of the IPSC reversal potential, reducing the strength of inhibition in SPN neurons, without changing the threshold for action potential firing. Regulating inhibitory strength, through cGMP-dependent changes in the efficacy of KCC2 in the target neuron provides a postsynaptic mechanism for rapidly controlling the inhibitory drive, without altering the timing or pattern of the afferent spike train. Therefore, this NO-mediated suppression of KCC2 can modulate inhibition in one target nucleus (SPN), without influencing inhibitory strength of other target nuclei (MSO, LSO) even though they are each receiving collaterals from the same afferent nucleus (a projection from the medial nucleus of the trapezoid body, MNTB).

## Introduction

The superior olivary complex (SOC) consists of groups of highly specialized brainstem nuclei that compute various acoustic features in sound location processing. Irrespective of the specific task, such as detecting differences in interaural time (medial superior olive; MSO), interaural intensity (lateral superior olive; LSO), or processing transient temporal information (superior paraolivary nucleus; SPN), glycinergic inhibition mediated by the medial nucleus of the trapezoid body (MNTB) is a key component of the afferent input to each of these nuclei (Grothe et al., 2010; Johnston et al., 2010). Thus, the MNTB is the major inhibitory hub within the SOC, with individual MNTB neurons providing collateral projections to multiple, functionally diverse targets (Banks and Smith, 1992; Sommer et al., 1993) where they serve fast and temporally precise inhibition, similar to the role of fast spiking inhibitory interneurons in other brain circuits (Bartos et al., 2002; Tepper et al., 2004). This common source of afferent inhibition (the MNTB) raises the question of what mechanisms are available to modulate the synaptic responses of the individual postsynaptic targets which serve differing roles in these diverse computational functions.

One key parameter of inhibitory strength is the chloride electrochemical gradient, which is the driving force for the hyperpolarizing action of synaptic inhibition and is determined by the intracellular chloride concentration. Early in development the sodium-potassium-chloride co-transporter type 1 (NKCC1) maintains a high intracellular chloride ([Cl^−^]_i_) concentration in most neurons and hence Cl^−^-mediated synaptic events are depolarizing (Cherubini et al., 2011; Friauf et al., 2011; but see Balakrishnan et al., 2003 for an exception). It is postulated that early in development, inhibitory synapses generate excitatory postsynaptic potentials (EPSPs) that act to stabilize synapse formation, and that as neurons mature there is a switch to expression of neuronal potassium chloride co-transporter type 2 (KCC2), driving a low [Cl^−^]_i_ and supporting hyperpolarizing IPSPs (Kandler and Gillespie, 2005). The trafficking, cell surface expression and transport-activity of KCC2 are closely controlled by neuronal activity (Fiumelli et al., 2005; Wake et al., 2007) with increased KCC2 activity caused by protein oligomerization and changes in phosphorylation (Casula et al., 2001; Blaesse et al., 2006; Chamma et al., 2012). As [Cl^−^]_i_ declines, the driving force favors influx of Cl^−^ ions which strengthens IPSPs and synaptic inhibition (Lohrke et al., 2005; Friauf et al., 2011; Ben-Ari et al., 2012). In mature neurons, KCC2 can be down-regulated under pathophysiological conditions, reducing the effectiveness of inhibition and causing hyperexcitability (Wake et al., 2007; Hewitt et al., 2009; Boulenguez et al., 2010). A variety of mechanisms modulate KCC2 activity and it is unclear which messengers mediate particular physiological responses, but one potential candidate is nitric oxide (NO).

NO is a gaseous messenger molecule involved in the regulation of synaptic transmission and neuronal function (Garthwaite, 2008). In neurons it is generated by neuronal nitric oxide synthase (nNOS) (Garthwaite and Boulton, 1995). Local NO may also reflect activity in blood vessels (from endothelial or eNOS) or inducible NO synthase (iNOS), with increased NO being associated with inflammatory and neurodegenerative diseases (Steinert et al., 2010; Nakamura et al., 2013) as well as following prolonged synaptic activity (Brenman et al., 1996; Holscher, 1997; Steinert et al., 2008, 2011). NO binds to its intracellular receptor, soluble guanylyl cyclase (sGC) leading to raised intracellular cGMP, which in turn interacts with multiple kinases and phosphatases (Francis et al., 2010).

Given the evidence for activity-dependent NO generation in neural cells (Garthwaite et al., 1988; Brenman et al., 1996; Steinert et al., 2008) and expression of KCC2 and nNOS in the SOC (Reuss and Riemann, 2000; Reuss et al., 2000; Balakrishnan et al., 2003; Lohrke et al., 2005; Blaesse et al., 2006) we asked if these signaling pathways might converge to adjust MNTB-mediated inhibition to the needs of the postsynaptic target neurons during specific stimulus conditions. Our results provide evidence that NO signaling can powerfully modulate the strength of inhibitory synaptic transmission by changing the Cl^−^ equilibrium potential via cGMP-dependent regulation of KCC2.

## Materials and methods

### *In vitro* preparations

All experimental procedures were approved by the Bavarian district government and were done according to the European Communities Council Directive (2010/63/EU). C57Bl6 mice and Mongolian gerbils (*Meriones unguiculatus*) (P12–P21) were killed by decapitation and coronal brainstem slices (200 μm-thick) containing the SOC were cut in a high sucrose, low-sodium artificial cerebral spinal fluid (ACSF) at ~0°C. Slices were maintained in a normal ACSF at 37°C for 30–45 min, after which they were stored at room temperature (~20°C) in a continually recycling slice-maintenance chamber. Composition of the normal ACSF was (mM): NaCl 125, KCl 2.5, NaHCO_3_ 26, glucose 10, NaH_2_PO_4_ 1.25, sodium pyruvate 2, myo-inositol 3, CaCl_2_ 2, MgCl_2_ 1, and ascorbic acid 0.5; pH was 7.4, bubbled with 95% O_2_, 5% CO_2_. For the low-sodium ACSF, NaCl was replaced by 200 mM sucrose, and CaCl_2_ and MgCl_2_ concentrations were changed to 0.5 and 6 mM, respectively. Experiments were conducted at physiological temperature with the recording chamber being continuously perfused with ACSF at a rate of 1–2 ml min^−1^. An inline feedback temperature controller and heated stage were used to maintain chamber temperature at 36 ± 1°C (TC344B, Warner Instruments, Hamden, CT, USA).

### Patch-clamp

Whole-cell patch-clamp and current-clamp recordings were made from visually identified SOC neurons (Olympus BX51WI microscope) using an EPC10/2 HEKA amplifier, sampling at 50 kHz and filtering at 10 kHz. Patch pipettes were pulled from borosilicate glass capillaries (GC150F-7.5, OD: 1.5 mm; Harvard Apparatus, Edenbridge, UK) using a DMZ Universal puller (Zeitz). Their resistance was ~3.5 MΩ when filled with a patch solution containing (mM): K-gluconate 97.5, KCl 32.5, HEPES 40, EGTA 5, MgCl_2_ 1, Na_2_phosphocreatine 5, pH was adjusted to 7.2 with KOH. Stated voltages are corrected for a liquid junction potential of −11 mV. Whole-cell series resistances were compensated by 50–80% and recordings in which the series resistance changed more than 2–3 MΩ were omitted from analysis. Synaptic currents were evoked by afferent fiber stimulation with a concentric bipolar electrode (FHC) driven by voltage pulses generated by the HEKA amplifier and post-amplified by a linear stimulus isolator (Pulse Stimulator AM-2100). Glutamatergic currents were blocked (50 μM D-AP5, 20 μM DNQX), GABAergic currents were blocked by 10 μM SR95531 and glycinergic currents were confirmed by blockade with 1 μM strychnine. The NO was applied via the NO donor sodium nitroprusside (SNP; 100 μM) which was prepared immediately before use. sGC was blocked with 1H-[1,2,4]Oxadiazolo[4,3-a]quinoxalin-1-one (ODQ, 1 μM).

### Immunocytochemistry

The brains of four Mongolian gerbils (*Meriones unguiculatus*) and four C57Bl6 mice (both aged 2–3 month) were perfusion-fixed with 4% paraformaldehyde, cryoprotected with 22.5% sucrose overnight and shock frozen in CO_2_ snow. Coronal brainstem sections were cut with a cryostat (40 μm thick; LEICA CM 3050S) and collected in phosphate buffered saline 0.05 M, pH 7.4 (PBS). After washing, non-specific binding sites were saturated with a blocking solution containing 1% BSA, 0.3% Triton X-100, and 0.1% saponin in PBS, for 1 h at room temperature and incubated in the primary antibody mix (diluted in blocking solution) for two nights at 4°C. The specificity of the primary antibodies used has been previously published for rodents and relevant publications are indicated for the respective antibodies. The primary antibodies used were: [rabbit anti-KCC2 1:500, Millipore, 07-432 (Kopp-Scheinpflug et al., 2011)], guinea pig anti-GlyT2 [1:500 Millipore, AB1773 (Hassfurth et al., 2010)], mouse monoclonal anti NOS-B1 [1:200, Sigma N2280 (Coote and Rees, 2008)], chicken anti-Map2 [1:1000, Neuromics, CH22103 (Kapfer et al., 2002)]. Subsequently, the sections were washed and incubated with the appropriate secondary antibodies: Alexa488 donkey anti-rabbit (1:300, Molecular Probes A21206), Cy3 donkey anti-guinea pig (1:300, Chemicon AP193C), Alexa488 donkey anti-mouse (1:300, Invitrogen A21202), Alexa 647 donkey-anti-chicken (1:300, Dianova 115-605-205). For the NADPH-d staining, sections were processed as described by Vincent and Kimura (1992).

### Image acquisition

**Figure 1:**
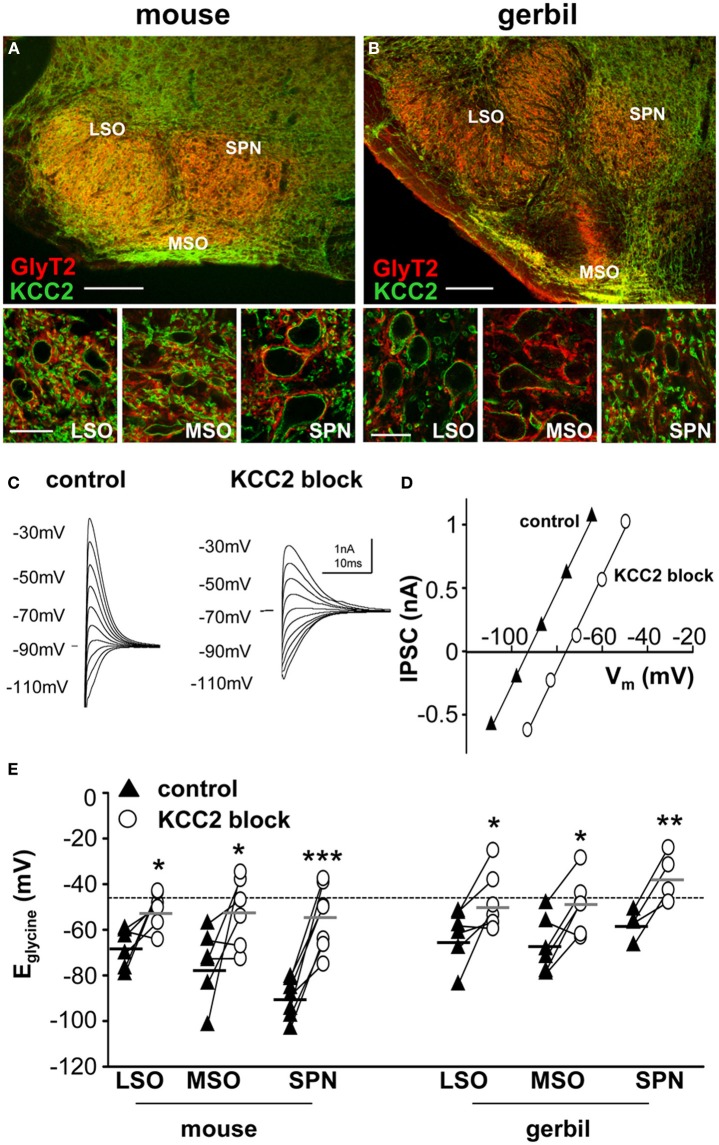
**KCC2 mirrors the expression of GlyT2 in the superior olivary complex**. Overview of mouse **(A)** and gerbil **(B)** SOC and enlarged images of LSO, MSO, and SPN double-labeled for GlyT2 (red), and KCC2 (green). Scale bars: 200 μm in overviews, 25 μm in magnified images. **(C)** Glycinergic IPSCs were evoked in a mouse SPN neuron by electrical stimulation of the MNTB. The command potentials ranged from −120 to −30 mV in steps of 10 mV. The IPSC reversal potential changed from −90 mV in control conditions (closed triangles in panel **D**) to −70 mV after the blockade of KCC2 (open circles in panel **D**) with furosemide. Stimulus artifacts were removed for clarity. **(D)** Current-voltage relationship for the IPSCs shown in **(C)**. The parallel shift of the curves indicates a sole change in reversal potential without changing the conductance. **(E)** IPSC reversal potentials were depolarized after blockade of KCC2. The deviations from the calculated reversal potential (black dotted line) are indicative of KCC2 activity levels and suggest that KCC2 is most active in the mouse SPN. The small black and grey horizontal lines represent the mean value of the respective control and the furosemide data sets. ^*^p ≤ 0.05, ^**^p ≤ 0.01 and ^***^p ≤ 0.001.

To image the NADPH-d stain in bright-field microscopy and to visualize immunohistochemical labeling, the sections were viewed with a VS120 S1 microscope [Olympus BX61VST with software dotSlide® (Olympus)]. For overviews in Figure 1A and in enlarged images of the immunohistochemical labeling, confocal optical sections were acquired with a Leica TCS SP confocal laser-scanning microscope (Leica Microsystems, Mannheim, Germany) equipped with a Plan 10.0×/NA 0.40 and a Plan 63×/NA1.32 oil immersion objective. After stack acquisition and Z chromatic shift correction between color channels, RGB stacks, montages of RGB optical sections, and maximum-intensity projections were assembled into tables using ImageJ (1.39q Wayan Rasband, National Institutes of Health, USA) and Adobe Photoshop CS6 (Adobe Systems, San Jose, CA) software; figure images were arranged using CorelDRAW X6 (Corel Corporation, Ottawa, Ontario, Canada).

### Measurement of KCC2 activity

Human neuroblastoma SHSY-5Y cells were grown in DMEM containing 10% fetal bovine serum at 37°C in a humidified atmosphere containing 5% CO_2_. Cells were seeded on 10 mm glass coverslips 48 h prior to the experiment. SHSY-5Y cells were loaded with the pH sensitive fluorescent dye BCECF-AM (0.5 μ M, 12 min. 2′,7′-bis-(2-carboxyethyl)-5-(and-6)-carboxyfluorescein acetoxymethyl ester; TefLabs) in a Ringer's solution (composition in mM: NaCl 120, MgCl 0.8, KCl 5.4, CaCl 1.8, HEPES 20, glucose 15) containing 0.1% BSA. Coverslips were placed in a perfusion chamber and imaged using 440/470 nm excitation filters and a bandpass emission filter at 535 nm (Chroma Technology) as described before (Hershfinkel et al., 2009). To monitor KCC2 activity we used the NH_4_Cl/BCECF paradigm (Hershfinkel et al., 2009). In absence of extracellular K^+^, NH_4_Cl (5 mM) added to the extracellular solution results in equilibrium between NH_3_ and NH^+^_4_. This is disrupted as NH_3_ rapidly diffuses through the membrane, binds H^+^ within the cytoplasm and causes alkalinization of the cells, which is detected by an increase in BCECF fluorescence (Figure 5A). In the absence of K^+^, the remaining extracellular NH^+^_4_ serves as a surrogate potassium ion and is transported into the cell by KCC2 via reverse transport (Chorin et al., 2011). The NH^+^_4_ influx changes the equilibrium between NH_3_ and NH^+^_4_ within the cells, releasing H^+^, and leading to acidification and a decrease of BCECF fluorescence. The rate of acidification is proportional to KCC2 activity, and blocking KCC2 in mature neurons blocks this transport and the acidification, leading to a prolonged alkalinization of the cells (Hershfinkel et al., 2009). Baseline KCC2 activity in SHSY-5Y cells is low, so the cells were initially incubated in low-osmolarity Ringer's solution containing only 100 mM NaCl for 5 min. Subsequently, baseline fluorescence was achieved and NH_4_Cl (5 mM) was added to the superfusion, nominally K^+^-free, Ringer's solution. The acidification rate, representing KCC2 activity, was calculated for a minimum of 20 cells/coverslip in each experiment. Rates were averaged across 5–10 independent measurements. As indicated, SNP (300 μM) was added for 45 min, at a concentration not toxic to these cells (Wagle and Singh, 2000) in the presence or absence of ODQ (1 μM) or the cell permeable Zn^2+^ chelator N,N,N′,N′-tetrakis (2-pyridalmethyl) ethylenediamine (TPEN, 10 μM). Statistical analyses were performed using analysis of variance with *post-hoc* comparisons.

### Data analysis and statistical methods

Data analysis was conducted with IgorPro 5.0 and custom written macros were employed. Statistical analyses of the data were performed with SigmaStat/SigmaPlot™ (SPSS Science, Chicago, IL). Results are reported as mean ± s.e.m.; *n* being the number of neurons recorded from at least three different animals. Statistical comparisons between different data sets were made using unpaired Student's *t*-test, while before and after comparisons were made by the paired Student's *t*-test. Differences were considered statistically significant at *p* < 0.05.

## Results

The neuronal glycine transporter type 2 (GlyT2) labels the inhibitory synaptic terminals around the respective target neurons, where it is responsible for the re-uptake of glycine from the synaptic cleft. Thus, GlyT2 is a reliable marker for neuron populations that receive strong glycinergic inputs. In the auditory brainstem, GlyT2 labeled neurons in the LSO, MSO, and SPN which all receive powerful inhibition from the MNTB (Figure 1). The labeling pattern of all three structures is comparable between mouse (Figure 1A) and gerbil (Figure 1B). The MSO is smaller in mouse compared to gerbil, as is expected for an animal with a small head and little low-frequency hearing. The immunohistochemistry also shows that KCC2 is expressed postsynaptically in the LSO, MSO and SPN of both mouse and gerbil and this mirrors the presynaptic labeling for GlyT2 (Figure 1 insets).

### KCC2 activity levels differ between brainstem nuclei

It is not possible to determine the activity and effectiveness of the KCC2 transporter in these different nuclei by measuring protein expression alone. KCC2 activity was monitored by measuring changes in the reversal potential of glycinergic synaptic currents evoked in response to electrical stimulation of the MNTB (and pharmacologically isolated by blocking glutamatergic and GABAergic components with 50 μM D-AP5, 20 μM DNQX, and 10 μM SR95531, respectively). The reversal potentials of the glycinergic currents, E_glycine_, were measured before (closed triangles in Figure 1C) and after blocking KCC2 activity with furosemide (0.5 mM; open circles in Figure 1C). In whole-cell patch recording, dialysis of the intracellular solution with the pipette solution enabled the introduction of a high intracellular chloride concentration (34.5 mM) that should maximize KCC2 activity in order to restore the usually low intracellular chloride concentration in LSO, MSO, and SPN neurons (Lohrke et al., 2005). The activity of KCC2 in these neurons is evident from the deviation of the measured IPSC reversal potential from the calculated reversal potential based on the internal and external chloride concentrations using the Nernst equation (E_calc_ = −46 mV; dotted line in Figure 1E). More negative deviations from E_calc_ toward more hyperpolarized reversal potentials provide an estimate for the activity of KCC2 that is driving this displacement. Blocking KCC2 by bath application of furosemide (0.5 mM) consistently shifted E_glycine_ toward more positive voltages near the calculated Nernst potential (Figure 1D). This shift was now measured for neurons in LSO, MSO and SPN in both mouse and gerbil (Figure 1E). Though there was a significant depolarizing shift in E_glycine_ for all three nuclei in both mouse and gerbil, the largest and most consistent shift in E_glycine_ was seen in the neurons of mouse SPN (Table 1).

**Table 1:** **Suppression of KCC2-activity measured as shift in E_glycine_ by 0.5 mM furosemide across different nuclei and species**.

	**Mouse**			**Gerbil**		
	**LSO**	**MSO**	**SPN**	**LSO**	**MSO**	**SPN**
Control E_glycine_	−68.6 ± 8.7 mV	−75.8 ± 15.7 mV	−90.6 ± 8.6 mV	−62.9 ± 4.9 mV	−67.4 ± 12.4 mV	−58.1 ± 3.03 mV
Furosemide E_glycine_	−51.0 ± 8.2 mV	−52.2 ± 15.4 mV	−54.5 ± 14.3 mV	−47.0 ± 13.7 mV	−49.0 ± 12.8 mV	−36.4 ± 5.4 mV
Δ E_glycine_	**17.6 mV**	**23.6 mV**	**36.2 mV**	**15.9 mV**	**18.4 mV**	**21.7 mV**
*n*	6	6	7	6	6	4
Significance	*p* = 0.037	*p* = 0.025	*p* ≤ 0.001	*p* = 0.035	*p* = 0.018	*p* = 0.014

### Nitric oxide as an additional (volume) transmitter in auditory brainstem signal processing

**Figure 2:**
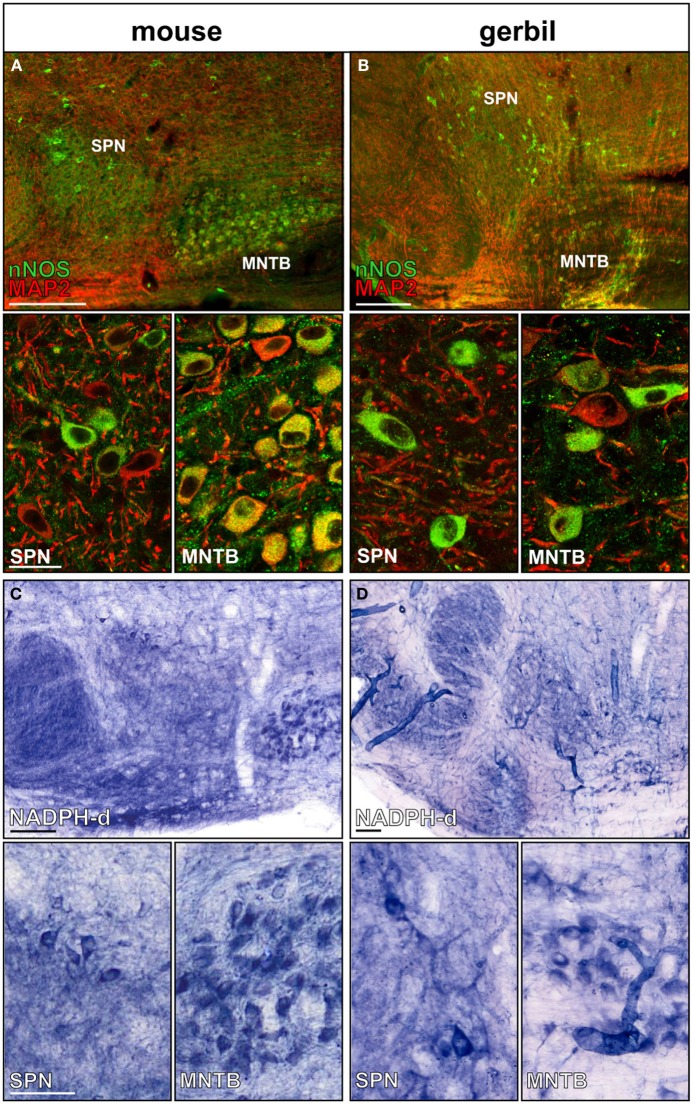
**nNOS expression in SPN and MNTB of mouse and gerbil**. Double-staining of **(A)** mouse and **(B)** gerbil SOC for MAP2 (red) and nNOS (green); insets show the respective high magnification images of SPN and MNTB. Histochemical staining of NADPH-diaphorase activity in the SOC of **(C)** mouse and **(D)** gerbil. High-magnification images of SPN and MNTB neurons show NADPH-d positive neurons in both nuclei confirming the nNOS staining. Scale bars: 200 μm in overviews, 25 μm in magnified images.

MNTB neurons express nNOS and generate the messenger molecule NO in an activity-dependent manner (Steinert et al., 2008, 2011). Here we show that nNOS is expressed in MNTB and SPN neurons of mice and gerbils (Figures 2A,B). Not all neurons in the SPN seem to express nNOS, but since NO acts as a volume transmitter it can affect even the nNOS negative neurons. Besides somatic nNOS staining, there is also a strong nNOS-positive labeling of the neuropil of the SOC nuclei. NADPH, a necessary coenzyme for the generation of NO has been used to successfully label nNOS positive neuronal somata as well as their axons (Luth et al., 1995; Reuss et al., 2000). Within the auditory brainstem NADPH positive somata are labeled in the MNTB and in the SPN (Figures 2C,D), corroborating the nNOS staining (Figures 2A,B). Similar to the nNOS staining there is heavily labeled neuropil in the LSO, MSO, and also the SPN, suggesting NO might be involved in local signal processing throughout the auditory brainstem. Although the labeling appeared weaker in the gerbil SOC this could reflect the lower specificity of the nNOS antibody that is based on the mouse sequence.

### NO signaling suppresses KCC2 activity in a cGMP dependent manner

**Figure 3:**
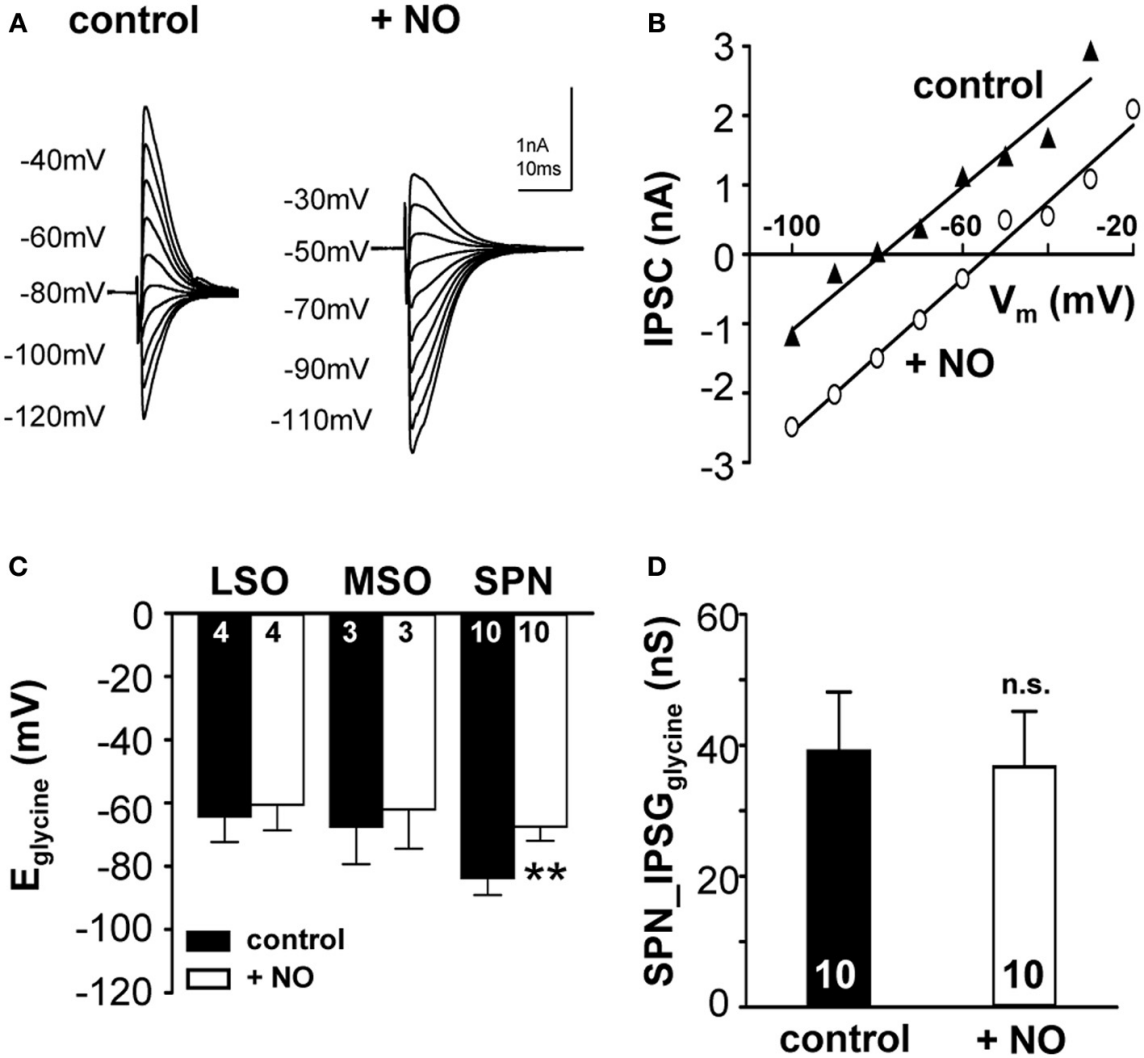
**Nitric oxide suppresses KCC2 activity**. **(A)** Glycinergic IPSCs were evoked in a mouse SPN neuron by electrical stimulation of the MNTB. The command potentials ranged from −120 to −30 mV in steps of 10 mV. The IPSC reversal potential changed from −80 mV in control conditions to −50 mV after the modulation of KCC2 activity by NO signaling. **(B)** Current–voltage relationship for the SPN-IPSCs shown in **(A)**. The parallel shift of the curves indicates a sole change in reversal potential without changing the conductance. **(C)** Average data show a significant depolarizing shift in IPSC reversal potential following NO application in the SPN, but not in LSO or MSO. **(D)** The overall glycinergic conductance in SPN neurons is unchanged by NO, indicating no change in the glycine receptor or the glycine release to be involved. ^**^p ≤ 0.01.

Sustained synaptic stimulation causes generation of NO within the SOC (Steinert et al., 2008, 2011). Here, endogenous NO release was mimicked by bath application of the NO-donor (SNP; 100 μM) and its effect on KCC2 activity was measured. Strong KCC2 activity drives the negative E_glycine_ (Lohrke et al., 2005) in mature mammalian auditory brainstem neurons. Within the nuclei of the SOC in mouse and gerbil, mouse SPN neurons showed the largest deviation from calculated reversal potential for glycine (Figure 1C), suggesting that KCC2 activity is strongest in the SPN. Therefore, SPN neurons might support an activity-dependent mechanism that allows down-regulation of KCC2, but this seems to be less essential in the MSO and LSO. To test this hypothesis, the effect of NO on KCC2 activity in mouse LSO, MSO and SPN was measured before and during the application of NO. NO did not affect KCC2 activity in MSO or LSO neurons, but caused a depolarizing shift in E_glycine_ from −83.7 ± 5.4 mV to −67.3 ± 4.5 mV (*n* = 10, *p* = 0.002; Figures 3A–C), in the SPN consistent with suppression of KCC2. The IPSC current-voltage relationship showed a parallel shift (Figure 3B) and the glycinergic conductance did not change significantly during the NO application (IPSG_control_: 39.1 ± 9.1 nS; IPSG_NO_: 36.7 ± 8.5 nS; *n* = 10; *P* = 0.57). These results indicate that there was no direct effect of NO signaling on the glycine receptors nor was there a major influence on presynaptic glycine release (Figure 3D).

**Figure 4:**
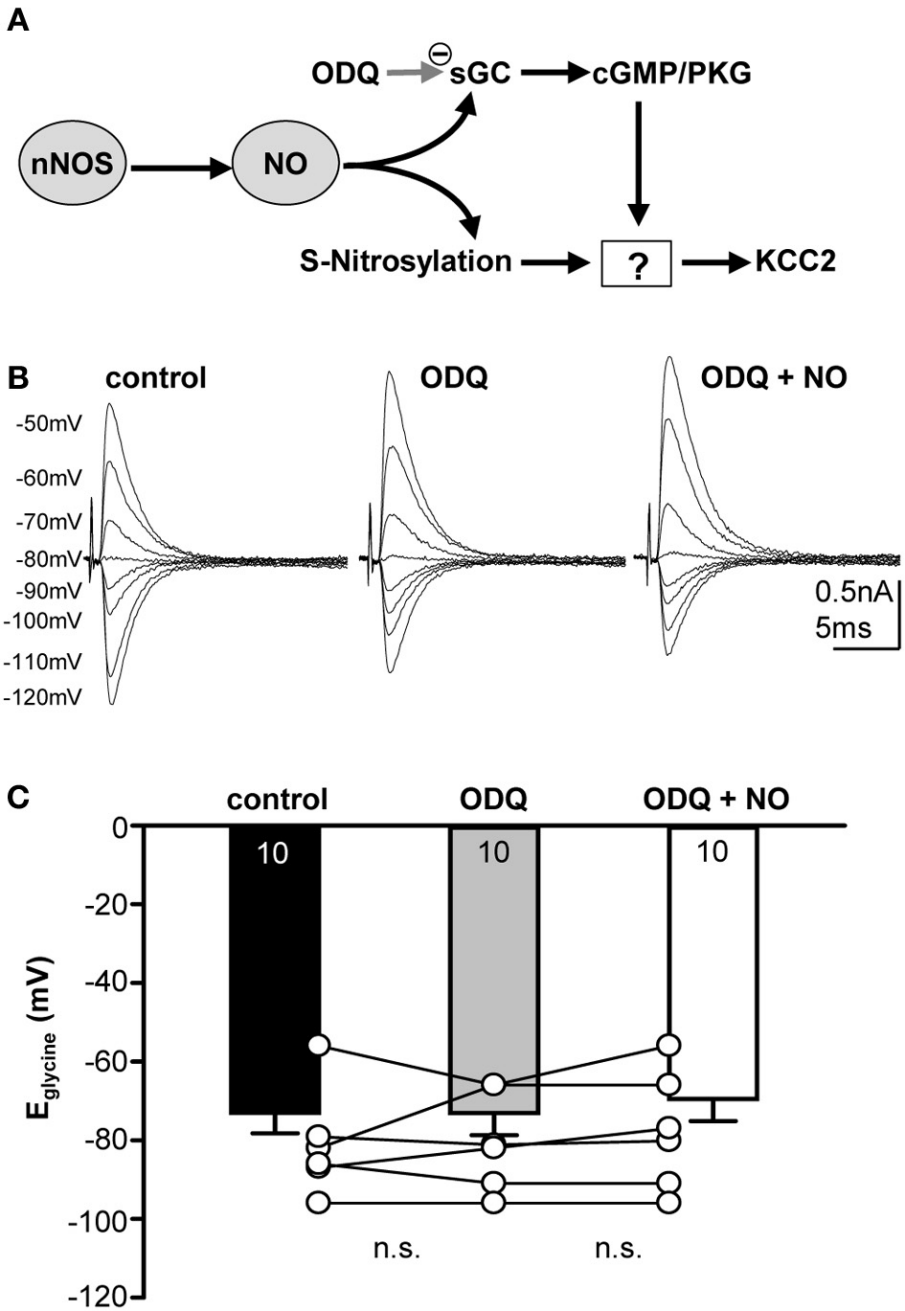
**Nitrergic signaling acting on KCC2 is cGMP-dependent. (A)** Cartoon of possible intracellular signaling mechanisms by NO. **(B)** Glycinergic IPSCs were evoked in a mouse SPN neuron by electrical stimulation of the MNTB. The command potentials ranged from −120 to −50 mV in steps of 10 mV. Blockade of sGC with ODQ prevents the depolarizing shift of the reversal potential normally observed following NO application. **(C)** Average data show that ODQ prevents the NO-mediated depolarizing shift in E_glycine_. There is no significant change between the three conditions.

The messenger molecule NO can mediate its action either via s-nitrosylation of proteins or by generation of cGMP and downstream activation of kinases or phosphatases (Figure 4A). To test between these mechanisms, ODQ (1 μM) was used to specifically block sGC. Indeed, the presence of ODQ in the bath resulted in stable E_glycine_ values even during the additional application of the NO-donor (Figures 4B,C; control-E_glycine_: −73.0 ± 5.2 mV; ODQ-E_glycine_: −73.1 ± 5.6 mV; ODQ/NO-E_glycine_: −69.5 ± 5.6 mV; *n* = 10; *P* = 0.809; ANOVA) suggesting that NO modulation of KCC2 is mediated via a sGC/cGMP-dependent signaling. We also analyzed the amplitudes during ODQ-conditioning and following perfusion of ODQ/NO. The change in amplitude was only about 10 percent and was not significant in ANOVA testing against the control condition (ODQ: 11 ± 8%; ODQ/NO: 9 ± 8%; *p* = 0.17).

**Figure 5:**
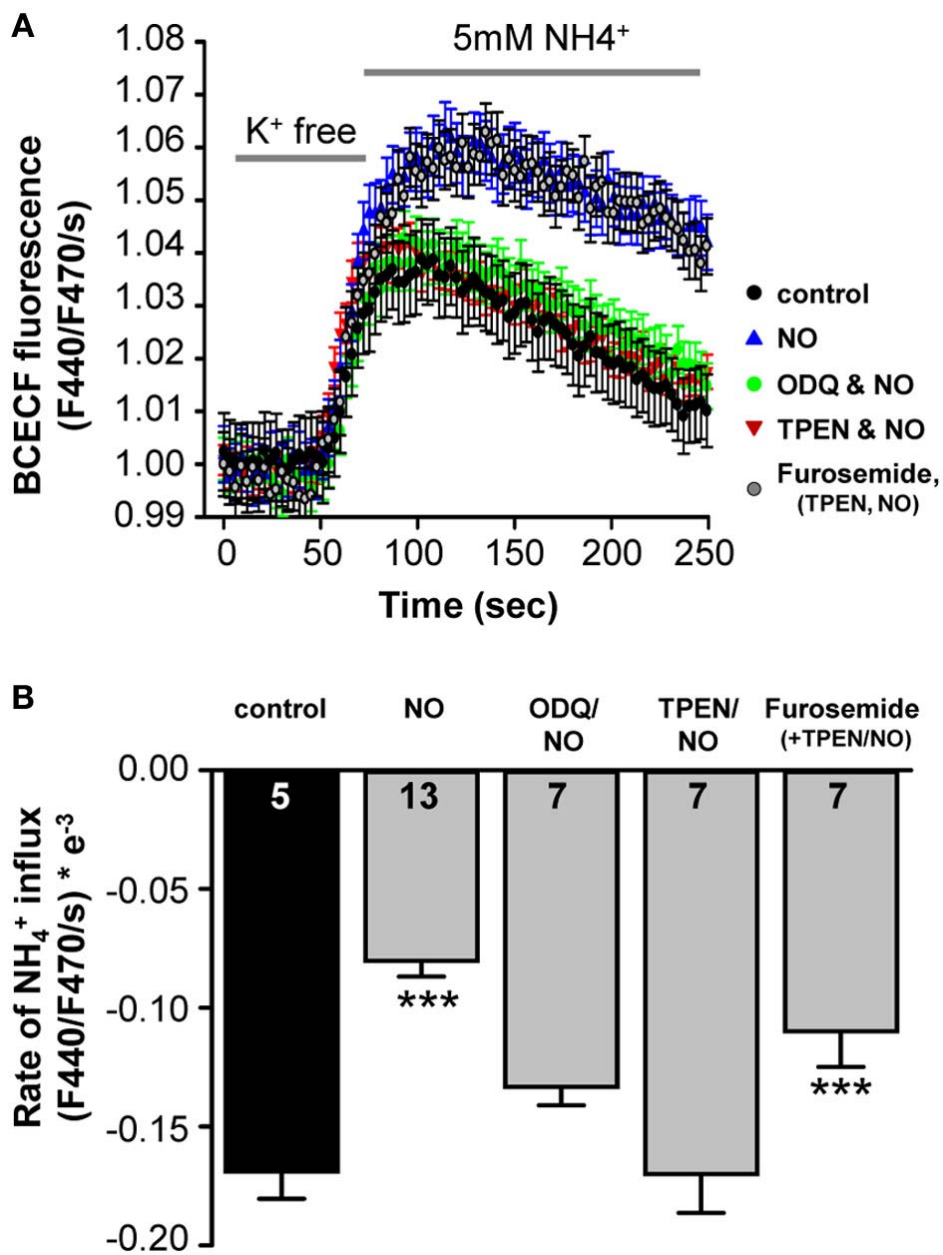
**NO-mediated suppression of KCC2 requires intracellular cGMP and Zinc**. **(A)** KCC2 activity was monitored with the pH-sensitive dye BCECF in SHSY-5Y cells that express endogenous KCC2. Cells were incubated for 5 min with K^+^ free extracellular solution, and the rate of intracellular pH change following application of NH_4_Cl (5 mM) was monitored (see Materials and Methods). **(A,B)** Plots of NH^+^_4_- mediated acidification rates (mean ± s.e.m.) in control, treated with NO, NO+ODQ, NO+TPEN, or ODQ+TPEN+furosemide. Significance was assessed using a One-Way ANOVA. Ns are given in the bars. ^***^p ≤ 0.001.

A direct test for NO regulation of KCC2, was achieved by monitoring KCC2-dependent NH^+^_4_ transport in SHSY-5Y neuroblastoma cells, which express endogenous KCC2 (Chorin et al., 2011). Using the NH_4_Cl paradigm, KCC2 activity is represented by acidification following reversed activity of KCC2 that is inducing NH^+^_4_ transport into the cells (see Materials and Methods). Under control conditions, NH^+^_4_-induced acidification rate of −1.7 ± 0.1 × 10^−4^ΔF_440_/F_470_/s (*n* = 5), was monitored (Figure 5A). Application of the NO-donor SNP (300 μM) resulted in down-regulation of KCC2 activity by about 2-fold to −0.80 ± 0.07 × 10^−4^ ΔF_440_/F_470_/s (*n* = 13; Figure 5B). This confirms that KCC2 activity is suppressed by NO (Figure 5B). In cortical neurons an increase in the intracellular zinc concentration has been shown to cause consistent KCC2-suppression (Hershfinkel et al., 2009). To test for a change in intracellular Zn^2+^ in the present experiment, a membrane permeable Zn^2+^ chelator (TPEN; 10 μM) was used to chelate intracellular Zn^2+^ prior to the application of SNP while KCC2-dependent NH^+^_4_-induced acidification rate was monitored in the cells. Interestingly, chelating intracellular Zn^2+^ completely blocked the effect of SNP (−1.7 ± 0.2 × 10^−4^ ΔF_440_/F_470_/s; *n* = 7), suggesting that increased concentrations of intracellular Zn^2+^ are involved in the NO-dependent attenuation of KCC2 activity in the SHSY-5Y cells (Figure 5B). To determine whether the increase in intracellular Zn^2+^ was caused by s-nitrosylation or by sGC/cGMP mediated signaling, the sGC inhibitor 1 μM ODQ was applied prior to and during the SNP and again the suppressive effect of the SNP was reversed to −1.3 ± 0.1 × 10^−4^ ΔF_440_/F_470_/s (*n* = 7), consistent with the hypothesis that the rise in [Zn^2+^]_i_ is downstream of sGC/cGMP/PKG in causing suppression of KCC2 (Figure 5B). Both, ODQ and also TPEN prevented the NO-mediated suppression of KCC2 (Figures 5A,B). If however, furosemide was applied in addition to ODQ and TPEN, KCC2 activity was again reduced due to a direct interaction between KCC2 and furosemide (Figures 5A,B).

### NO suppresses IPSPs and offset firing without changing intrinsic action potential threshold

**Figure 6:**
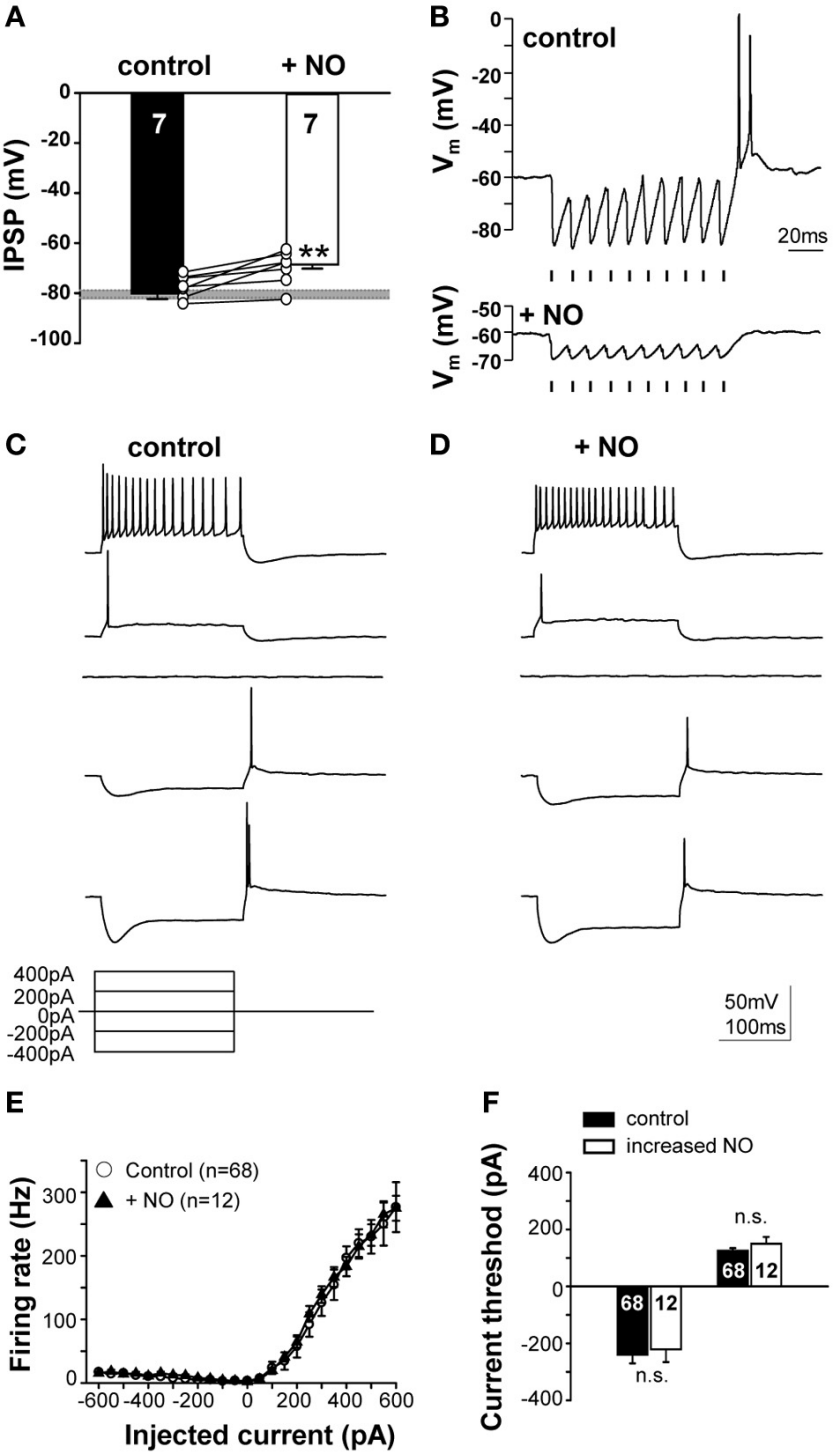
**NO suppresses IPSPs and offset firing without changing intrinsic action potential threshold**. **(A)** Suppression of KCC2 causes synaptically-evoked IPSPs to be less hyperpolarizing. The grey bar indicates the voltage threshold for generating rebound action potentials (−80.4 ± 1.5 mV; *n* = 71). **(B)** Glycinergic IPSPs in response to 100 Hz pulse trains (indicated by the mark below the recordings) evoked rebound action potentials at the end of the train in the control condition (upper trace). Application of NO (lower trace) reduced the IPSP amplitudes and did not result in rebound firing. Stimulus artifacts were removed for clarity. **(C)** Current-clamp responses to hyperpolarizing and depolarizing current injections revealed similar firing patterns in the control and **(D)** the NO condition. **(E)** Input–output functions did not differ between control and NO conditions. **(F)** Injection of similar magnitude currents evoked similar rebound firing (or firing in response to depolarization) for both conditions, indicating no significant change in action potential threshold. ^**^p ≤ 0.01.

NO mediated suppression of KCC2 reduces the IPSP-driven membrane hyperpolarization from −80.1 ± 2.2 mV to −68.6 ± 1.6 mV (*n* = 7; *p* ≤ 0.001; Figure 6A). The typical offset/rebound firing pattern of SPN neurons in response to sound requires the evoked IPSPs to hyperpolarize the membrane potential to about −80 mV (Kopp-Scheinpflug et al., 2011). Following NO signaling, inhibitory synaptic inputs will no longer generate rebound firing (Figure 6B). The SPN firing response to MNTB-evoked IPSP trains was compared in the presence of low (control) or high NO (Figure 6B). In control conditions, the trace shows a burst of offset action potentials at the end of the stimulus train, but after raising NO in the test condition, only smaller IPSPs were generated which did not trigger offset firing at the end of the train.

This NO-mediated change in SPN firing could reflect a change in postsynaptic intrinsic excitability, as observed in the MNTB (Steinert et al., 2008), so somatic injection of hyperpolarizing and depolarizing currents were used (Figure 6C) to test neuronal excitability. The mean rate-level functions measured under control conditions completely overlapped with that measured during the raised NO condition, indicating that there was no significant change in intrinsically evoked action potential firing. Action potential number and current threshold were the same before and during application of NO (Figures 6C–F). This result corroborates the finding that NO-mediated suppression of KCC2 is the mechanism mediating the change in the neuronal firing in the SPN, rather than any direct action of NO on action potential generation.

### Physiological relevance: no reduces gap-detection ability of SPN neurons

**Figure 7:**
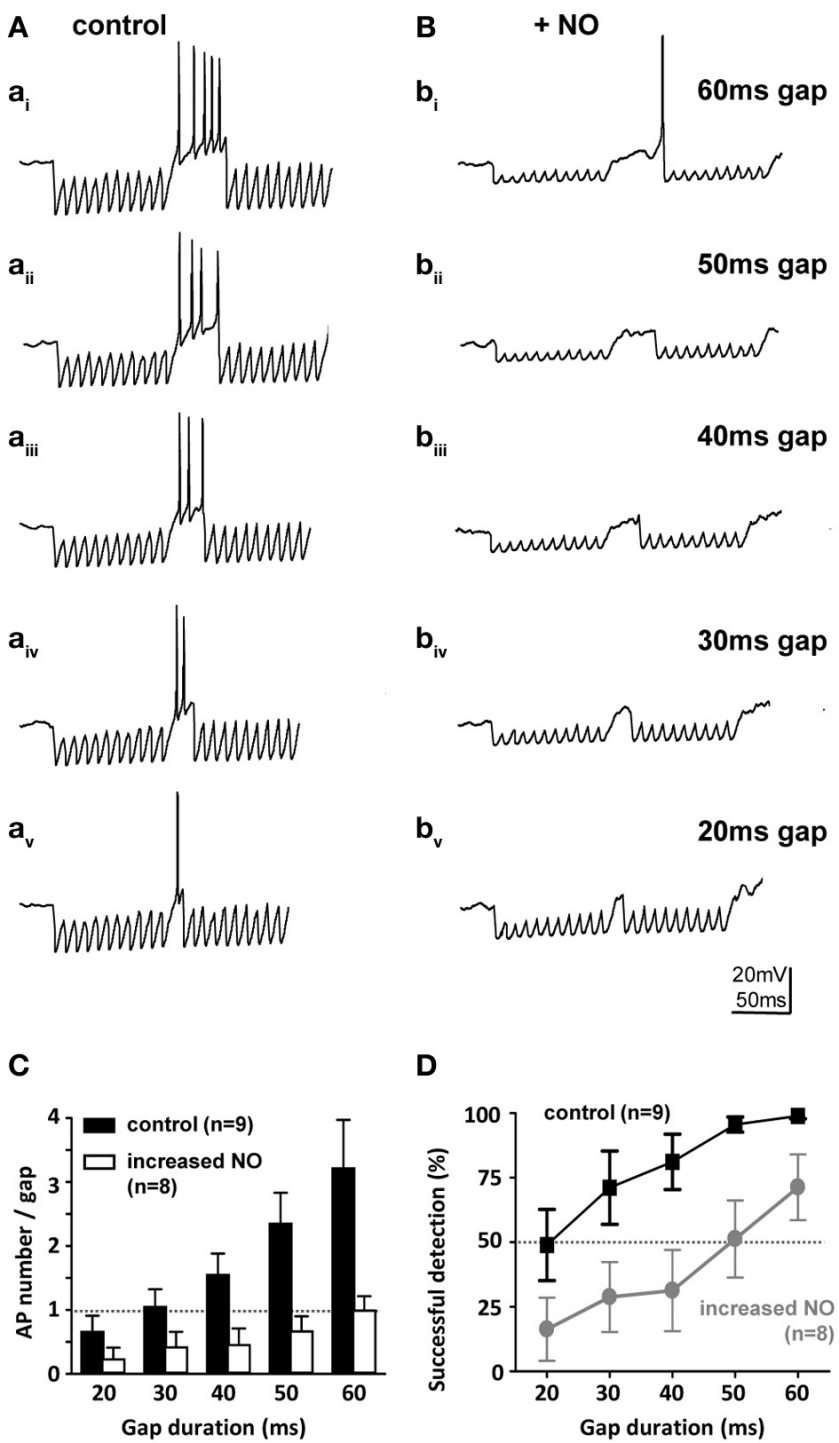
**Nitric oxide suppresses gap-detection on a cellular level**. **(A)** Mouse SPN neurons show offset firing in responses to gaps of different durations (60–20 ms) embedded in trains of electrically evoked IPSPs. **(B)** Bath application of an NO donor reduced the number of action potentials per gap and delayed the action potential within the gap so that only longer gaps were detected. Stimulus artifacts were removed for clarity. **(C)** The number of evoked action potentials increased with gap duration in controls (black bars), but was never more than one with NO (white bars). **(D)** Repetitive stimulation (10 IPSP trains) was used to estimate gap-detection success, which is plotted here as % offset action potentials against gap duration. Threshold was defined as 50% success (dashed line); controls (black) reliably detected gaps of 20 ms or longer, but following NO, gap-detection thresholds increased to 60 ms or longer. Data plotted as mean ± s.e.m. (*n* indicated on the respective graph).

SPN neurons receive a range of synaptic projections and express a suite of voltage-gated ionic conductances that enable these neurons to integrate their inputs and fire rebound action potentials at the end of an IPSP train (Kopp-Scheinpflug et al., 2011). This in turn allows computation of auditory gap-detection (Kadner and Berrebi, 2008). We used a gap-detection paradigm to explore how this physiological mechanism is influenced by NO signaling. Gap-detection on a cellular level was determined by current-clamp recording from SPN neurons during synaptic stimulation of the inhibitory inputs from the MNTB. Two 100 Hz stimulus trains of 100 ms duration each were separated by gaps of 20, 30, 40, 50, or 60 ms (each gap-protocol was repeated 10 times). At the gap, short-latency offset action potentials were generated (Figure 7A) in the SPN neurons, with action potential numbers proportional to gap-duration (Figure 7C). In the control condition (low-NO), all gaps evoked action potentials and gaps of 20 ms or longer were reliably detected with success rates greater than 50%. However, following bath application of NO, gap-detection thresholds increased, so that only longer gaps triggered action potentials (Figures 7B,C) and gap-detection for durations shorter than 60 ms was disabled (Figure 7D).

## Discussion

The MNTB is a common source of glycinergic inhibition, but this innervation is performing different functions in each of the target nuclei. Given that individual MNTB neurons are projecting to multiple nuclei and firing patterns are the same for each target, it is important to understand how the inhibitory synaptic strength can be independently modulated in each target nucleus to fine-tune local synaptic actions. Conventionally two options are considered: presynaptic modulation of transmitter release and postsynaptic modulation of receptor activation and/or receptor kinetics. Here we demonstrate a third option—namely modulation of the IPSC reversal potential. Our results show that the gaseous messenger molecule NO can serve as a control device of inhibitory synaptic strength by controlling E_Cl_/E_glycine_ in the postsynaptic target neurons via cGMP-dependent suppression of the potassium-chloride co-transporter type 2 (KCC2).

KCC2 drives a low internal chloride concentration, hence increasing the hyperpolarizing action of inhibition. KCC2 is present throughout the central nervous system, but is particularly highly expressed in the hippocampus, hypothalamus, brainstem, and motor neurons of the spinal cord (Vinay and Jean-Xavier, 2008; Blaesse et al., 2009). Developmental up-regulation of KCC2 expression strengthens hyperpolarizing inhibition (Cherubini et al., 2011; Friauf et al., 2011). Consequently, acquired loss of KCC2 function in mature neurons will lead to hyperexcitability and seizures due to less hyperpolarizing inhibitory inputs (Wake et al., 2007; Vinay and Jean-Xavier, 2008; Boulenguez et al., 2010; Arion and Lewis, 2011). Deficient KCC2 activity has been described following neuronal damage such as physical trauma or ischemia and the following mechanisms are suggested to be involved: transcriptional regulation via neurotrophin receptor activation (Rivera et al., 2002, 2004), post-translational regulation via changes in the phosphorylation state of KCC2 (Blaesse et al., 2009; Chamma et al., 2013) and activity-dependent down-regulation after NMDA-receptor activation and calcium influx (Ginsberg, 2008; Lee et al., 2011). Previous experiments in the auditory brainstem show that activation of NMDA receptors in the MNTB causes the necessary calcium influx that triggers the activation of nNOS and thus the generation of NO (Steinert et al., 2008). As a volume transmitter NO can then act on the surrounding neurons of LSO, MSO, and SPN. The SPN itself also expresses nNOS [Figure 2; (Reuss, 1998; Reuss et al., 2000)], providing a further local source of NO in the SPN. Here, we studied the link between NO and the mechanism by which KCC2 is down-regulated. Our present data show that NO-mediated KCC2 suppression is absent if sGC is blocked, suggesting that KCC is suppressed via a cGMP-dependent mechanism (Figure 4). An additional possible downstream mechanism is the NO-mediated recruitment of intracellular Zn^2+^ that suppresses KCC2 activity (Hershfinkel et al., 2009). NO can trigger increase in [Zn^2+^]_i_ in at least two different ways; either via cGMP/PKG-dependent release from internal stores (Jang et al., 2007) or via s-nitrosylation of metallothioneins (Zhang et al., 2004). Our ODQ-data support the NO-cGMP pathway leading to Zn^2+^-mediated suppression of KCC2.

In the central nervous system NO is generally thought to act as a retrograde volume transmitter that modulates transmitter release presynaptically (for a recent reviews see Hardingham et al., 2013). Here we show a postsynaptic mechanism which mediates a parallel shift in the IPSC current-voltage relationship that is inconsistent with a presynaptic change in transmitter release. Instead we demonstrate that this regulation in inhibitory strength is mediated by modulation of the chloride equilibrium potential and does not involve the glycine receptors directly. It is interesting to note that this mechanism is likely to be unique to inhibitory synaptic transmission, since changing the sodium or potassium equilibrium potential would interfere with action potential generation, propagation and waveform.

### Significance of KCC2 suppression for information processing in the SPN

The present study employs a mouse model for *in vitro* demonstration of NO-mediated regulation of sound offset encoding/gap-detection mechanisms in the auditory brainstem. Brief gaps in sound determine sound rhythms (Felix et al., 2011; Kopp-Scheinpflug et al., 2011) and suggest a correlation between gap-detection and speech perception (Snell et al., 2002; Frisina et al., 2006). Although information about sound offsets and gaps is processed in the auditory cortex, it is derived from subcortical computations (Scholl et al., 2010). Gaps in sound are reliably encoded in the SPN in the auditory brainstem (Kadner and Berrebi, 2008; Kopp-Scheinpflug et al., 2011) by a mechanism that requires three elements: large glycinergic IPSPs, driven by an extreme negative chloride reversal potential (E_Cl_), combined with a large hyperpolarization-activated non-specific cationic current (I_H_), and a T-type calcium conductance (I_TCa_) (Kopp-Scheinpflug et al., 2011). Our present data show that NO negatively modulates KCC2 which can lead to the following cascade of events: less hyperpolarized IPSPs cause less activation of I_H_ (HCN channels) and do not provide sufficient hyperpolarization to enable recovery of I_TCa_ (low-threshold calcium channels) from inactivation; therefore the typical offset firing/gap detection in SPN neurons is restricted by NO. The loss of temporal resolution (as measured by gap-detection) is a prevalent dilemma in models of ageing, hearing loss and neurodegeneration. At the same time individuals with inflammatory and neurodegenerative diseases often show increased levels of NO in nervous system tissue (Sweeten et al., 2004; Steinert et al., 2010). Effective modulators of gap-detection thresholds would provide an important mechanism which would influence higher levels of processing. Attempts to improve gap-detection by facilitating GABAergic inhibition have failed (Gleich and Strutz, 2011) which is consistent with the *glycinergic*/KCC2 mechanism as described here. Facilitation of KCC2 (e.g., by NO-scavengers) could be considered for future treatments as a means to enhance local inhibition in the brain.

### Significance of KCC2 suppression for binaural information processing in the MSO and LSO

In both, the interaural level difference processing LSO as well as in the interaural time difference processing MSO, the balance of excitation and MNTB-mediated inhibition is crucial for adjusting the binaural sensitivity of single neurons as well as the population output (Grothe et al., 2010). Traditionally considered to be rather static, the binaural sensitivity of both, LSO and MSO, has recently been shown to be surprisingly dynamic (Magnusson et al., 2008; Hassfurth et al., 2010; Fischl et al., 2012; Stange et al., 2013). Modulation via NO may superimpose these adaptations on the binaural system by globally adjusting synaptic sensitivity to changing levels of acoustic exposure. However, according to our present study, NO-mediated suppression of KCC2 activity results in a weakening of the main inhibitory input to SPN neurons. In contrast, KCC2 activity in LSO and MSO neurons is not affected, suggesting a different role of NO than suppressing inhibition.

In summary, our results show that the NO synthesizing enzyme nNOS is expressed in the SOC where NO can be generated in an activity-dependent manner. Future studies should aim at identifying the significance of NO as a volume transmitter in the auditory brainstem in an *in vivo* preparation. NO can act as a powerful modulator of inhibitory transmission by suppressing KCC2. This novel mechanism of activity-dependent modulation of the equilibrium potential could be widely utilized in other areas of the nervous system to control local inhibitory strength.

## Author contributions

Lina Yassin: conducted electrophysiological experiments and analyzed data, Susanne Radtke-Schuller: conducted immunohistochemistry, Hila Asraf: conducted KCC2 measurements, Benedikt Grothe: interpreted data and jointly wrote manuscript, Michal Hershfinkel: designed KCC2 measurements, interpreted data and jointly wrote manuscript, Ian D. Forsythe: conceived project jointly with Cornelia Kopp-Scheinpflug, interpreted data and jointly wrote manuscript. Cornelia Kopp-Scheinpflug: conceived project, conducted experiments, analyzed and interpreted data, wrote manuscript.

### Conflict of interest statement

The authors declare that the research was conducted in the absence of any commercial or financial relationships that could be construed as a potential conflict of interest.
